# A Study to Evaluate the Potential Role and Clinical Application Value of Long Noncoding RNA CASC Family Members in Colorectal Cancer Based on Transcriptomic Data

**DOI:** 10.1155/ijog/3881424

**Published:** 2025-07-24

**Authors:** Chao Liang, Jun Wang, Xinyu Liu

**Affiliations:** ^1^Department of General Surgery, The First Affiliated Hospital of Jinzhou Medical University, Jinzhou, China; ^2^Department of Geriatrics, The First Affiliated Hospital of Jinzhou Medical University, Jinzhou, China

**Keywords:** cancer susceptibility candidate, colorectal cancer, diagnosis, long noncoding RNAs, protein network

## Abstract

**Background:** Long noncoding RNA (lncRNA) CASC, crucial in colorectal cancer (CRC) progression, remains largely unexplored despite its potential.

**Methods:** The CRC data comes from The Cancer Genome Atlas (TCGA) database. The limma package was used to screen differentially expressed genes (DEGs), intersecting with CASC genes that yielded key hub lncRNAs. Next, the lncRNA–protein interaction network was developed applying Cytoscape software. The association between immune cell infiltration and lncRNAs was calculated using the ESTIMATE package, CIBERSORT package, and ssGSEA. Based on the survminer package to assess the correlation between hub gene expression levels and clinicopathologic features of CRC patients, cellular models were utilized to assess the mRNA expression levels and potential biological functions of the screened markers.

**Results:** We filtered 2326 DEGs that were notably enriched in pathways related to metastasis, cell growth, and EMT. This study found six hub lncRNAs (CASC15, CASC16, CASC8, CASC9, CASC19, and CASC18) showed a high diagnostic accuracy, with the area under the curve (AUC) values all exceeding 0.7. There were 44 proteins in the lncRNA–protein interaction network that interact with hub lncRNAs, among which both LIN28B and IGF2BP2 interact with six hub lncRNAs. Immune infiltration analysis indicated that the six hub lncRNAs were significantly correlated with the multiple types of immune cells. Pathological analysis demonstrated that the expression of CASC15 elevated with the progression of TNM staging. Cellular assays had revealed that all are significantly associated with CRC; particularly, CASC15 knockdown repressed the in vitro metastasis of CRC cells.

**Conclusion:** We constructed and validated a robust signature of six lncRNA CASC for predicting survival of CRC patients and characterizing the immune infiltration landscape. These results reveal that the CASC gene family could be a therapeutic target for CRC patients.

## 1. Introduction

Colorectal cancer (CRC) ranks as the third most frequently detected cancer and the second primary cause of cancer mortality globally [1], with around 1.8 million newly diagnosed cases and around 881,000 deaths reported worldwide in 2018 [2, 3]. The number of existing cases and deaths is projected to reach 2.2 million in 2030, according to statistical analysis modeling [4–6]. Similar to treatment options for other cancers, surgery, chemotherapy, radiation, and targeted therapies are the current modalities for treating CRC. Drug resistance and metastatic CRC, however, are among the major barriers to cure [7]. Metastatic disease accounts for the majority of CRC-related deaths, and resistance to standard chemotherapy limits the effectiveness of systemic therapy, often leading to relapse and disease progression [8–10]. In addition, breakthroughs in knowledge about immune system research have made immunotherapy an increasingly important treatment option [11, 12]; the recurrence rate has reached more than 40% [13]. Although immunotherapy has improved the survival rate of CRC, ineffective activation of tumor-specific immune responses could be caused by impaired processing and presentation of neoantigens, a lack of suitable neoantigens, and epigenetic modifications [14]. Metastatic CRC remains an important cause of incomplete cure [15]. Due to the lack of early diagnostic means of CRC, 45% of patients have metastasis by the time of diagnosis [8]. Therefore, more accurate and effective molecular diagnostic tools need to be discovered urgently.

The cancer susceptibility candidate (CASC) family gene cluster consists mainly of a series of long noncoding RNAs (lncRNAs) that fulfill crucial biological functions in the genesis, progression, and metastasis of a wide range of tumors [16]. CASC2, CASC9, CASC15, and CASC11 are important CASC signature genes that have been found to promote CRC disease progression. CASC9 plays a critical role in CRC carcinogenesis, and it was found that inhibition of CASC9 function can suppress the proliferative and migratory abilities of CRC cells by enhancing cellular autophagy and attenuating the activity of the AKT/mTOR/EMT signaling pathway in the cells [17]. CASC2 may be a target of berberine in the treatment of CRC, and knockdown of CASC2 can reverse apoptosis of CRC cells caused by berberine [18]. The expression of CASC15 in CRC cells was remarkably elevated, and overexpression of CASC15 can lead to CRC cell growth and invasion [19]. In a study by Zhang et al., it was noted that increased expression of CASC11 in CRC was associated with tumor size, serum infiltration, lymphatic metastasis, and tumor node metastasis (TNM) stage, and it was also noted in their study that CASC11 interacted with hnRNP-K to activate the WNT/beta-catenin pathway, which promotes CRC growth and metastasis [20]. These studies illustrate that CASC-related genes are biologically important in CRC, and revealing their functions can help to enhance the survival rate of CRC. However, the number of CASC studies in CRC is low, and numerous genes with unknown functions still need to be explored.

In this study, we comprehensively analyzed CASC-characterized lncRNAs in CRC and identified key hub lncRNAs by differential expression analysis and functional enrichment analysis. This prognostic feature provided a novel direction for the early diagnosis, individualized treatment, and prognostic evaluation of CRC with potential clinical applications.

## 2. Methodology

### 2.1. Dataset Sources

Expression data from the CRC sequencing program and clinical randomization data (TCGA-COAD_READ) were sourced through The Cancer Genome Atlas (TCGA, https://portal.gdc.cancer.gov/) database. The expression data in TCGA-COAD_READ were processed in two ways. (1) FPKM values of the RNA expression data therein were converted to TPM and then log2-transformed. (2) Samples with incomplete records of survival time and survival status were removed and deleted from the cohort for analysis. After processing, those that met the requirements for analysis included 582 CRC samples and 51 paraneoplastic control samples. In addition, a search for CASC-associated lncRNAs in the GeneCards database using the keyword “Cancer Susceptibility Candidate” yielded 14 CASC-associated lncRNAs, including CASC2, CASC15, CASC16, CASC11, CASC8, CASC22, CASC9, CASC17, CASC19, CASC20, CASC21, CASC23, CASC18, and CASC6.

### 2.2. Differential Expression Analysis and Functional Enrichment Analysis

The expression profiles of CRC samples and paracancerous samples were differentially analyzed by R package limma in TCGA-COAD_READ for the identification of differentially expressed genes (DEGs) therein, with screening thresholds of |log2FC| > log2 (1.2) and *p* < 0.05 [21, 22]. We performed a functional enrichment analysis by the LNCSEA2.0 online tool (https://bio.liclab.net/LncSEA/index.php) to investigate the potential functions of DEGs, and the pathway with *p* < 0.05 was considered biologically significant [23, 24].

### 2.3. Hub lncRNAs Associated With CASC and Evaluation

Taking the intersection of DEGs in CRC with 14 CASC-associated lncRNAs, the overlapping lncRNAs among them were used as hub lncRNAs for CASC features in CRC. The receiver operating characteristic (ROC) curves of hub lncRNAs were plotted by pROC package to explore the diagnostic performance of the molecular hub lncRNAs [25]. In addition, principal component analysis (PCA) was performed on the samples using the expression profiles of hub lncRNAs as features.

### 2.4. Constructing the lncRNA–Protein Interaction Network

Proteins with potential interactions with hub lncRNAs were obtained by LNCSEA2.0 (http://bio.liclab.net/LncSEA/index.php) tool. The lncRNA–protein interaction network was constructed by Cytoscape software with interaction pairs with confidence level > 0.7, and the results were visualized [26].

### 2.5. Correlation Analysis Between Hub lncRNAs With Immune Cells/Immune Pathways

The StromalScore, ImmuneScore, and TumorPurity [27] of CRC samples were calculated by the estimate package in TCGA-COAD_READ. The relationship between StromalScore, ImmuneScore, and TumorPurity and the expressions of hub lncRNAs was then assessed based on the Spearman correlation analysis method. Using the R package CIBERSORT [28], we calculated the infiltration of 22 common immune cell types in the dataset samples, which represent the infiltration of immune cells. This process relies on the LM22 signature provided by the CIBERSORT website (https://cibersortx.stanford.edu/), which encompasses expression data for 22 common immune infiltrating cell types. Applying the single-sample GSEA (ssGSEA) function in the GSVA package [29], the infiltration of immune cells in CRC patients and controls was quantified and performed enrichment analysis on 28 immune cell types [30].

In addition, the HALLMARK pathway was downloaded through the MSigDB (https://www.gsea-msigdb.org/gsea/index.jsp) database, and ssGSEA was performed through the GSVA package to calculate the ssGSEA score of the HALLMARK pathway [29, 31]. The same Spearman correlation analysis–based method was used to assess the correlation between the expressions of hub lncRNAs and the ssGSEA score of the HALLMARK pathway.

### 2.6. Correlation Analysis of Hub Genes With Clinicopathologic Features

In TCGA-COAD_READ, CRC samples were categorized into low and high expression groups by the R package survminer using the expressions of hub lncRNAs to determine the optimal cutoff value [32]. Survival analysis was performed in both groups to assess the potential impact of hub lncRNAs on progression-free survival. Further, in different TNM subgroups, we evaluated the expression levels of hub lncRNAs to assess their potential impact on CRC pathologic staging.

### 2.7. Cell Culture and Transfection

Human normal colon mucosal epithelium cell line NCM460 (SNL-519) and CRC cell line SW1116 (SNL-448) were ordered from Sunncell (Wuhan, China) and cultured in Roswell Park Memorial Institute-1640 medium (SNM-001A, Sunncell) and Leibovitz's L15 medium (SNM-007A, Sunncell). All these media were added with 10% bovine calf serum (SNS-002, Sunncell) and 1% penicillin–streptomycin (SNA-001, Sunncell). An incubator of a routine condition (37°C with 5% CO_2_) was used for all cell culture.

For the transfection, CASC15-specific small interfering RNA (target sequence: 5⁣′-GGGGAAATGTCCCTTAAAAGTGC-3⁣′) and the control small interfering RNA with random sequence were all ordered from RiboBio (Guangzhou, China) and transfected into CRC cell SW1116 using Lipofectamine 3000 transfection reagent (L3000-001, Invitrogen, Carlsbad, California, United States) as per the manuals.

### 2.8. Cell Migration and Invasion Determination

For wound healing assay, CRC cells transfected with CASC15-specific small interfering RNA or the control small interfering RNA were reseeded in six-well plates at 1 × 10^5^/well. When cells became fully confluent, a sterile pipette tip was used to create the wounds. Following the 48-h culture, the cell debris was removed via washing in PBS, and the remaining cells were observed under an inverted optical microscope (GX71, Olympus Corp., Tokyo, Japan).

Additionally, for transwell assay, transfected CRC cells were seeded into upper transwell chamber (3422, Corning Inc., Corning, New York, United States) containing the serum-free culture media (200 *μ*L) coated with Matrigel matrix (M8370, Solarbio Lifesciences, Beijing, China), while the corresponding lower compartment was filled with 700 *μ*L complete culture media. Following the culture for 48 h, cells remaining in the upper chamber were removed with a cotton swab, while those invaded into the lower compartment were sequentially fixed in 4% paraformaldehyde (P1110, Solarbio Lifesciences, China) for 15 min and dyed in 0.1% crystal violet (G1063, Solarbio Lifesciences, China) for 10 min. The same optical microscope was used to observe the invaded cells [33]. Finally, the results of the cell migration and invasion assays were calculated using ImageJ software.

### 2.9. Quantitative Reverse-Transcription PCR (qRT-PCR)

TRIzol reagent (15596026, Invitrogen, United States) was applied to extract the total RNA from CRC cell SW1116 and normal human colon mucosal epithelial cell NCM460, following which the cDNA was synthesized via a commercial kit (6110A, Takara Bio, Shiga, Japan). Subsequently, the cDNA was applied for the PCR using a relevant assay kit (204243, Qiagen, Hilden, Germany) in Mx3000 Real-Time PCR System (Agilent, Santa Clara, California, United States) at the following conditions: 95°C for 15 min and 40 cycles of 94°C for 15 s, 60°C for 30 s, and 72°C for 30 s. The 2[-delta delta c(t)] quantification method was applied for the calculation of the mRNA levels with GAPDH as the housekeeping control [34]. The primers involved in this assay are available in Table S1.

### 2.10. Statistical Analyses

R software (Version 4.1.1) and GraphPad Prism software (Version 8.0.2) were employed for the bioinformatics and the statistical analyses, respectively. For bioinformatics, the Wilcoxon test was applied for two-group comparison, while the Kruskal–Wallis test was used to calculate significant differences between multiple groups. Student's *t*-test was applied in the comparison of data in two groups for cellular assays. The threshold of statistically significant difference was set when *p* value was lower than 0.05.

## 3. Results

### 3.1. DEGs in CRC and Their Functions

**Figure 1:**
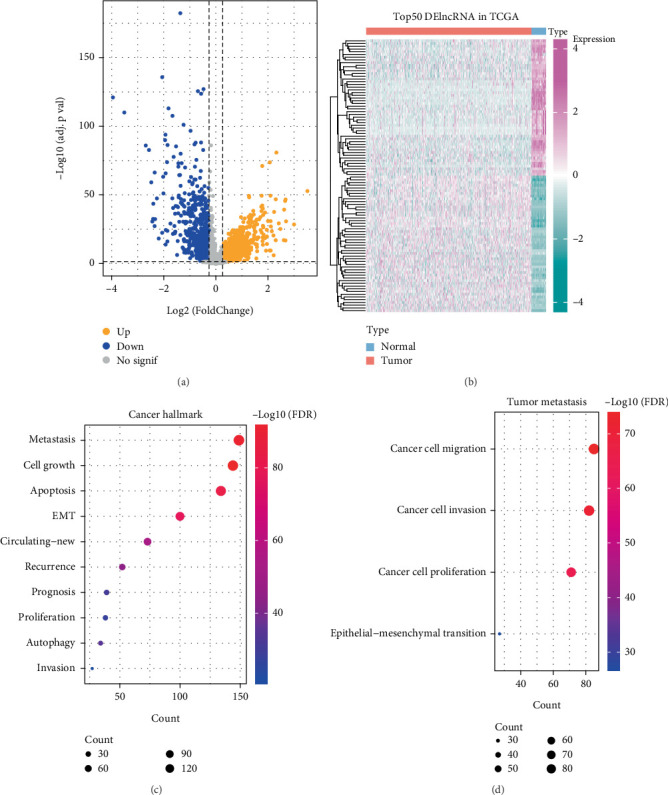
DEGs in CRC and their functions. (a) Volcano diagram showing DEGs in TCGA-COAD_READ. (b) Heatmap demonstrating DEGs in TCGA-COAD_READ. (c) Bubble plot demonstrating the cancer hallmark–associated pathway involved in highly expressed DEGs. (d) Bubble plot demonstrating the tumor metastasis–related pathway involved in highly expressed DEGs.

In the TCGA-COAD_READ dataset, the expression profiles of lncRNAs in 51 paraneoplastic samples and 582 CRC samples were differentially analyzed. There were 2326 DEGs in both, including 1572 upregulated DEGs and 754 downregulated DEGs (Figure 1a,b). The 1572 upregulated DEGs were subsequently analyzed for functional enrichment. Evidently, we intuitively noticed that the upregulated DEGs were mainly involved in tumor metastasis, cell growth–related pathways, metastasis, cell growth, apoptosis, EMT (Figure 1c), cell migration, invasion, and proliferation, and epithelial–mesenchymal transition (Figure 1d).

### 3.2. Hub lncRNAs Associated With CASC and Evaluation

**Figure 2:**
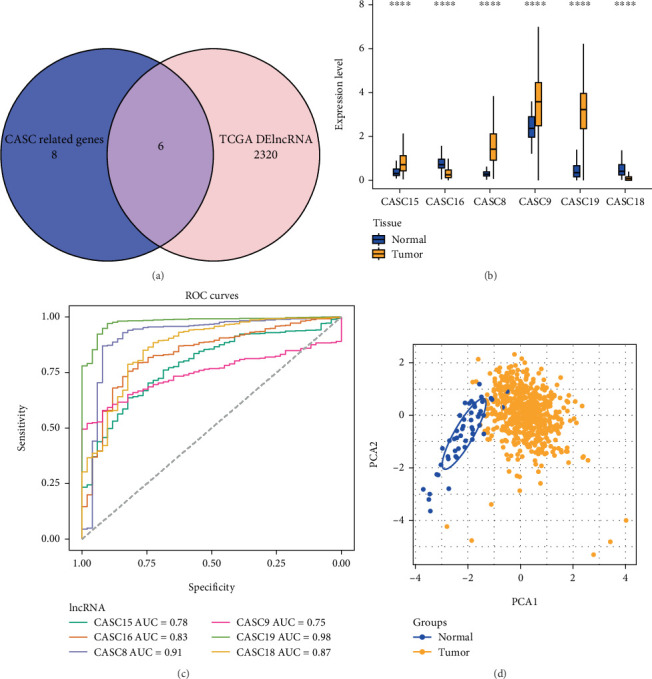
CRC diagnostic model composed of six hub lncRNAs. ∗∗∗∗ represents *p* < 0.0001. (a) Venn diagram identifying hub lncRNAs characterized by CASC in CRC. (b) Box plots of expression levels of CASC15, CASC16, CASC8, CASC9, CASC19, and CASC18 in paraneoplastic and CRC samples in the TCGA-COAD_READ cohort. (c) ROC curves of CASC15, CASC16, CASC8, CASC9, CASC19, and CASC18 diagnosed CRC. (d) Scatterplot of principal component analysis based on the expression profiles of six hub lncRNAs.

Taking the intersection of 2326 DEGs with 14 CASC-associated lncRNAs, there were six overlapping lncRNAs, namely, CASC15, CASC16, CASC8, CASC9, CASC19, and CASC18, which we thought might be the hub lncRNAs for CASC characterization in CRC (Figure 2). Meanwhile, we noticed that CASC15, CASC8, CASC9, and CASC19 were highly expressed in tumor samples and CASC16 and CASC18 were lowly expressed in tumor tissues (Figure 2). Further analysis of the diagnostic efficacy of the individual hub genes, with AUC values greater than 0.7. The AUC values of CASC15, CASC16, CASC8, CASC9, CASC19, and CASC18 for the diagnosis of CRC were 0.78, 0.83, 0.91, 0.75, 0.98, and 0.87, respectively (Figure 2). In the TCGA-COAD_READ cohort, the samples were subjected to PCA based on the expression profiles of six hub lncRNAs, and the results demonstrated that the CRC samples and the paraneoplastic samples displayed a clear boundary (Figure 2).

### 3.3. lncRNA–Protein Interaction Network in CRC

**Figure 3:**
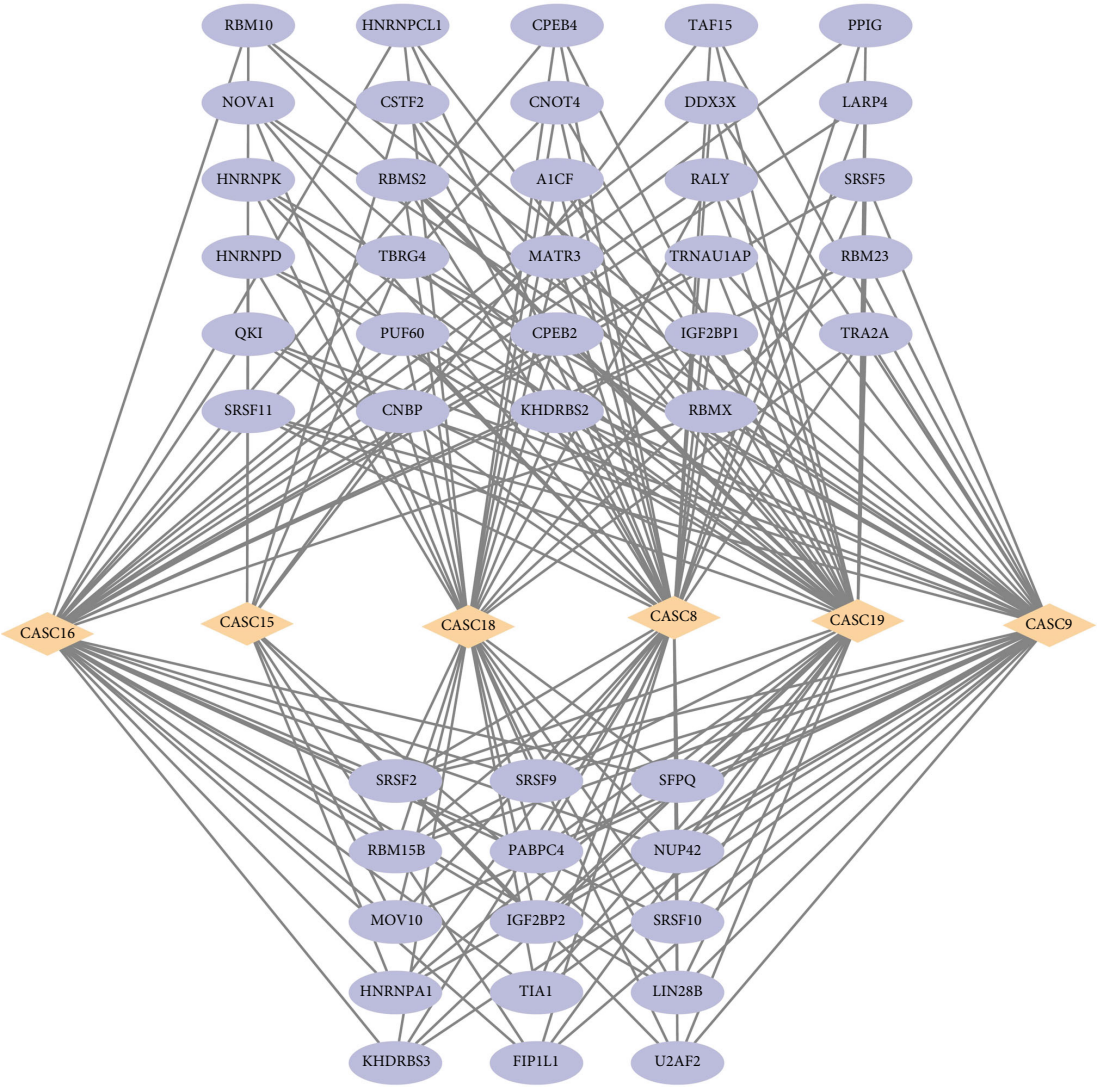
lncRNA–protein interaction network in CRC.

We obtained proteins that have interactions with six hub lncRNAs from the LNCSEA2.0 database and used Cytoscape to build the lncRNA–protein interaction network for visualization. We observed 44 proteins in the network that interacted with the six hub lncRNAs (Figure 3). Upon closer inspection of the network, each protein interacts with at least three hub lncRNAs. Particularly, lin28b and igf2bp2 are two of the most important proteins, which interact with CASC15, CASC16, CASC8, CASC9, CASC19, and CASC18.

### 3.4. Correlation Analysis Between Immune Infiltration and Six Hub lncRNAs in CRC

**Figure 4:**
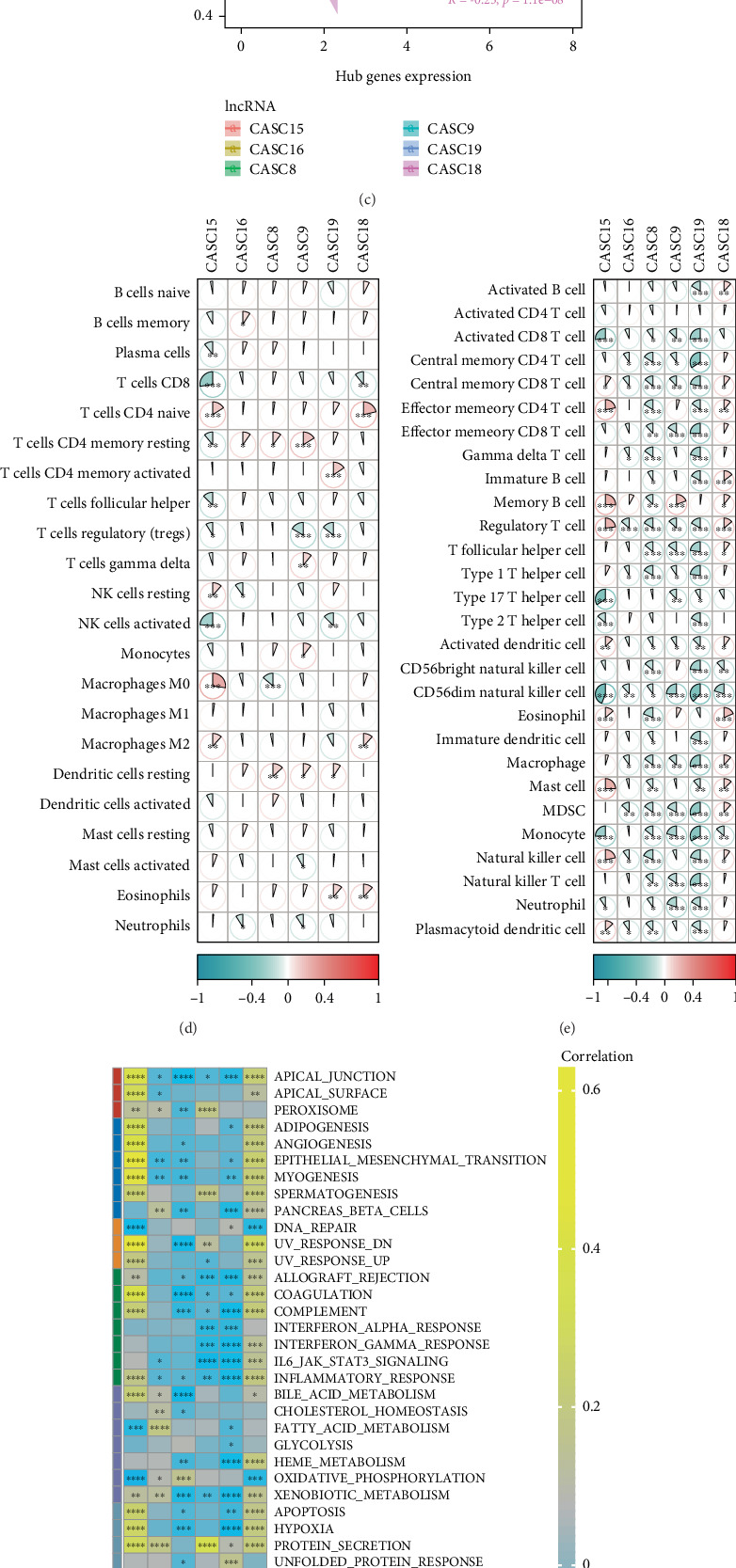
Correlation analysis of six hub lncRNAs with immune infiltration score in CRC. (a) Correlation of six hub lncRNAs with StromalScore. (b) Correlation of six hub lncRNAs with ImmuneScore. (c) Correlation of six hub lncRNAs with TumorPurity. (d) CIBERSORT was used to calculate the correlation analysis between six hub lncRNAs and 22 types of immune cells. (e) ssGSEA was used to calculate the correlation analysis between six hub lncRNAs and 28 types of immune cells. (f) Correlation of six hub lncRNAs with HALLMARK pathway ssGSEA score. In the graphs, ∗ indicates *p* < 0.05, ∗∗ indicates *p* < 0.01, ∗∗∗ indicates *p* < 0.001, and ∗∗∗∗ indicates *p* < 0.0001.

In the TCGA-COAD_READ dataset, we calculated StromalScore and ImmuneScore as well as TumorPurity in CRC and then further assessed the correlation of six hub lncRNAs with them. CASC18 (*R* = 0.27, *p* < 0.05) and CASC15 (*R* = 0.45, *p* < 0.05) were significantly positively correlated with StromalScore (Figure 4). Nonetheless, these 6 hub lncRNAs did not show a very strong correlation with ImmuneScore (Figure 4). CASC18 (*R* = −0.23, *p* < 0.05) and CASC15 (*R* = −0.29, *p* < 0.05) were significantly negatively linked to TumorPurity (Figure 4). An analysis was conducted on the relationship between six hub lncRNAs and immune cell infiltration. The CIBERSORT immune results showed that these six hub lncRNAs were closely related to most immune cells, with the highest correlation observed with T cells CD4 memory resting (Figure 4, *p* < 0.05). Meanwhile, the ssGSEA also indicated significant correlations between most immune cells and these six hub lncRNAs, particularly central memory CD8 T cell, myeloid-derived suppressor cells (MDSCs), regulatory T cell, Macrophage, activated dendritic cell, CD56dim natural killer cell, monocyte, and natural killer cell, which exhibited significant correlations with five or more lncRNAs (Figure 4, *p* < 0.05).

In addition, we calculated the ssGSEA scores of HALLMARK pathways and assessed the correlation of six hub lncRNAs with them. CASC15 presented significant positive correlation with most cancer-related pathways, TGF_BETA_SIGNALING, NOTCH_SIGNALING, WNT_BETA_CATENIN_SIGNALING, ANGIOGENESIS, and PI3K_AKT_MTOR_SIGNALING (Figure 4f). These results imply that the key lncRNAs we identified may play important regulatory roles in CRC progression, metastasis, and immune escape.

### 3.5. Correlation of Six Hub lncRNAs With Clinicopathologic Features of CRC

**Figure 5:**
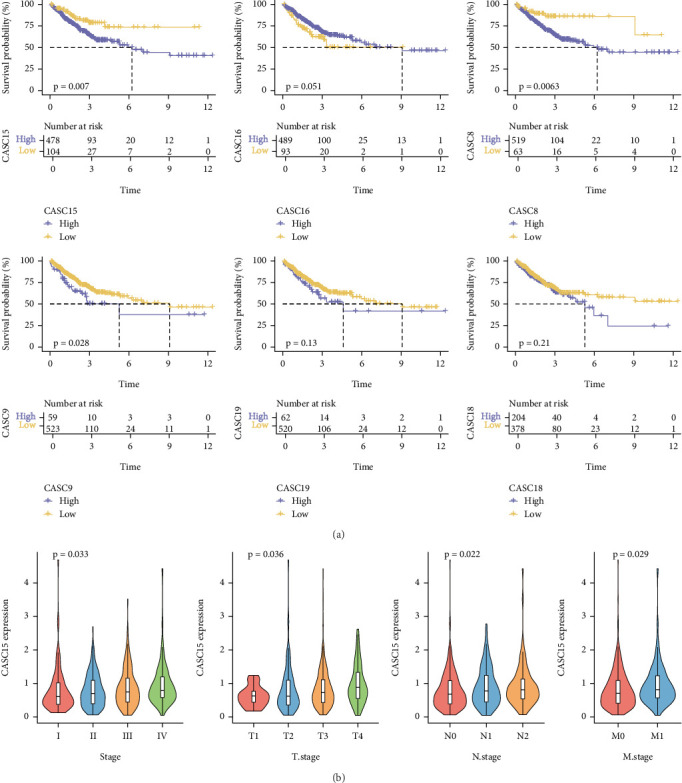
Correlation of six hub lncRNAs with clinicopathologic features of CRC. (a) Progression-free survival curves of six hub lncRNAs in CRC. (b) Expression levels of CASC15 in different TNM stages.

**Figure 6:**
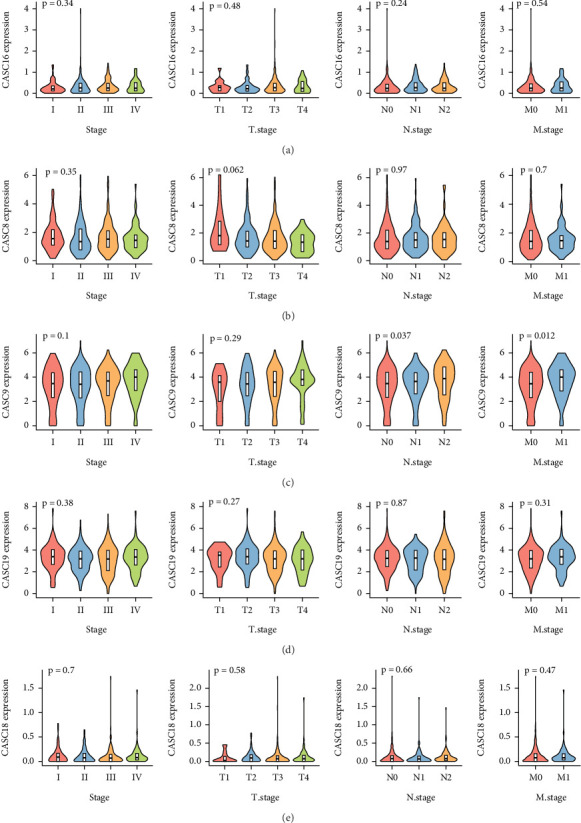
Correlation of six hub lncRNAs with TNM staging in CRC. (a) Expression levels of CASC16 in different TNM stages. (b) Expression levels of CASC8 in different TNM stages. (c) Expression levels of CASC9 in different TNM stages. (d) Expression levels of CASC19 in different TNM stages. (e) Expression levels of CASC18 in different TNM stages.

Samples were assigned into low and high expression groups according to the expression level of six hub lncRNAs using the optimal threshold determined by the survminer package as a cutoff value, and survival analyses were performed in both high and low expression groups. We noted that high expression of CASC15, CASC8, and CASC9 in CRC resulted in poorer progression-free survival (Figure 5a). Finally, we explored the correlation between six hub lncRNAs and clinical features in tumors and found that the expression of CASC15 was elevated with the progression of TNM staging (Figure 5b), whereas the correlation between CASC16, CASC8, CASC9, CASC19, and CASC18 and clinicopathological features was not significant (Figures 6a, 6b, 6c, 6d, and 6e).

### 3.6. Validation on Six Hub lncRNAs in CRC Cells

**Figure 7:**
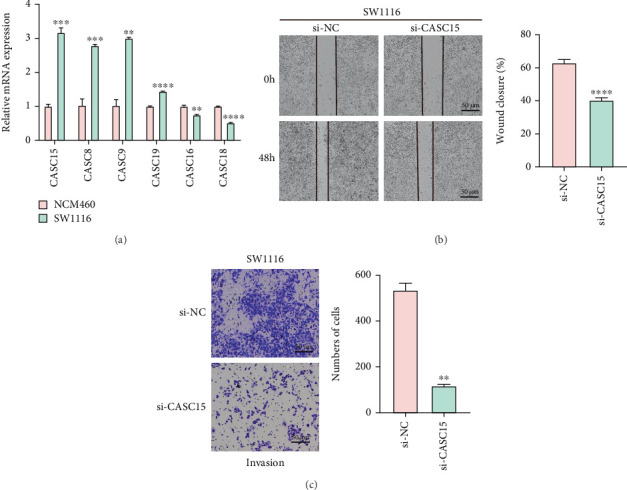
Validation of the screened key hub lncRNAs in CRC cell lines. (a) Expression levels of these hub lncRNAs in CRC cell SW1116 and normal colon mucosal epithelial cell NCM460. (b) Effects of CASC15 silencing on the migration of CRC cell SW1116. (c) Effects of CASC15 silencing on the invasion of CRC cell SW1116. In the graphs, ∗∗ indicates *p* < 0.01, ∗∗∗ indicates *p* < 0.001, and ∗∗∗∗ indicates *p* < 0.0001.

Finally, we sought to explore the potential involvement of these six hub lncRNAs in CRC, and their levels in CRC cell SW1116 were accordingly quantified. As compared to the normal colon mucosal epithelial cell NCM460, higher expression of CASC15, CASC8, CASC9, and CASC19 yet lower expression of CASC16 and CASC18 in CRC cells were observed (Figure 7a, *p* < 0.01). Additionally, relevant results from wound healing and transwell assays have underlined that CASC15 knockdown repressed the in vitro migration and invasion of CRC cells (Figure 7b,c, *p* < 0.01).

## 4. Discussion

Accumulated data have demonstrated that CRC is a serious life-threatening malignancy, and the lack of awareness of early screening has led to increasing patient mortality [6, 35, 36]. With the upgrading of biotechnology, the oncogenic role of CASC-related genes has become clearer [37]. CASC9 was found to activate mTOR-dependent autophagy and EMT pathway activity in CRC to promote cancer progression [17]. The interaction between CASC9 and CPSF3 can also exert oncogenic activity by regulating the TGF-beta pathway in the body [38]. These results suggested that CASC-related genes played a crucial role in the development of CRC. However, so far, CASC-related genes in CRC have been less studied, and there is still a broad scientific prospect. Therefore, the discovery of more comprehensive and precise early diagnostic markers and prognostic markers is essential to improve the survival of patients suffering from CRC [39].

In this study, six CASC-characterized hub lncRNAs, CASC15, CASC16, CASC8, CASC9, CASC19, and CASC18, were identified in CRC. In fact, the literature had found that these CASC15, CASC8, CASC9, and CASC19 lncRNAs are all closely related to the occurrence of CRC. Among them, upregulation of CASC15 expression levels in CRC could activate the EMT pathway which in turn led to a poor progression-free survival [40]. This was consistent with our finding that, in our results, we clearly observed that CASC15 expression in tumor tissues was notably higher than in paraneoplastic tissues and that CASC15 contributed to the poor progression-free survival of CRC. Alternatively, CASC15 exhibited a role as a sponge. Upregulated CASC15 expression in CRC cells promoted LGR5 expression by inhibiting miR-4310 expression, a state that activates the Wnt/*β*-catenin signaling pathway in CRC to promote cancer cell metastasis, migration [41]. In addition, studies had also found that CASC15 can directly inhibit the expression of miR-145 in HT29/OXA and HCT116/OXA [42]. Since miR-145 may be related to chemotherapy resistance in a variety of malignant tumors, this was also a potential reason for CASC15 to affect the prognosis of CRC [43]. A study noted that the QTL locus in CASC8 (rs6983267) was a risk factor for CRC, and people with this genotype may be more likely to be diagnosed compared to normal individuals [44]. Knockdown of CASC9 could competitively bind miR-576-5p to regulate AKT3 expression to inhibit CRC cell proliferation [45]. Evidence showed that overexpressed CASC19 elevated the expressions of CEMIP and EMT biomarkers in CRC cells, which can be reversed by inducing the expression of miR-140-5p in cells [46]; they were highly correlated with the poor cancer prognosis [47]. However, CASC16 and CASC18 functions had not been reported in CRC, and their functions need to be further explored. In addition, six hub lncRNAs also showed excellent diagnostic performance in CRC, with diagnostic AUC values all higher than 0.7. These studies suggested that they may be hub lncRNAs for CASC characterization in CRC, with the potential to be used as diagnostic biomarkers for CRC.

We constructed the hub lncRNA–protein interaction network and identified LIN28B and IGF2BP2 as two of the most important proteins, which interacted with multiple hub lncRNAs, including CASC15, CASC16, CASC8, CASC9, CASC19, and CASC18. These interactions suggest potential regulatory roles in CRC progression. Studies demonstrated that LIN28B and IGF2BP2 are highly linked to CRC occurrence and progression. LIN28B promotes cell invasion and metastasis through pathways involving CLDN1 and NOTCH3 [48]. Additionally, LIN28B contributes to CRC tumor growth through the LIN28B/IRS1 axis, a target of miR-30a-5p, which promotes tumor proliferation [49]. These findings emphasize LIN28B's role in CRC progression, particularly in cell invasion, tumor growth, and signaling regulation. Furthermore, emerging evidence links LIN28B to the CRC immune microenvironment. Studies suggest that LIN28B promotes cell survival and tumorigenesis by increasing BCL-2 expression and inhibiting apoptosis, indicating a potential influence on the tumor microenvironment (TME) [50]. This influence may extend to modulating the immune response and shaping immune cell activity in CRC. Similarly, IGF2BP2 is crucially involved in CRC progression, particularly through its regulation of iron metabolism via upregulating TFRC expression [51]. The analysis of leukocyte subsets indicated that the high expression of IGF2BP2 was highly negatively related to the infiltration of tumor-infiltrating lymphocytes (TILs) [52]. Previous studies had also found that the expression of IGF2BPs showed a significant positive correlation with the tumor infiltration of macrophages, B cells, and CD8^+^ T cells, and it affected tumor growth and immune responses within TME-associated macrophages [53, 54]. Overall, LIN28B and IGF2BP2 not only play key roles in promoting CRC cell proliferation, invasion, and metastasis but may also be involved in the process of remodeling the immune microenvironment of CRC by modulating immune-related pathways and influencing immune cell infiltration. This suggests that both may serve as potential targets for regulating TME and immunotherapy response.

Moreover, the interactions between these proteins and the lncRNAs suggest possible mechanisms through which they modulate CRC immune responses, such as affecting immune cell infiltration and activation within the TME. Further studies are warranted to elucidate the precise regulatory pathways and to explore their potential as therapeutic targets, particularly in modulating the CRC immune microenvironment. Through pathway analysis of CAS15 enrichment, it was found that CAS15 is significantly positively correlated with most tumor-related pathways such as TGF_BETA_SIGNALING, PI3K_AKT_MTOR_SIGNALING, NOTCH_SIGNALING, WNT_BETA_CATENIN_SIGNALING, and ANGIOGENESIS. Among them, the Wnt/*β*-catenin signaling pathway is an evolutionarily highly conserved signaling pathway. Embryonic development and adult homeostasis can be controlled by the Wnt family of secreted glycolipoproteins through signal transduction of the transcriptional coactivator *β*-catenin [55] and affects the occurrence of CRC [56]. Studies have found that lncRNA CASC15 can cause *β*-catenin to enter the nucleus in large quantities through the Wnt pathway, promoting EMT in cancer cells [57]. TGF-*β* is a major cytokine classified as an EMT inducer and is associated with the highly invasive phenotype of cancer [58]. The PI3K/AKT/mTOR signaling pathway fulfills crucial biological functions in cell growth, proliferation, apoptosis, autophagy, angiogenesis, and other processes [59]. Studies have shown that all three major subfamilies of the PI3K/Akt/mTOR signaling pathway may be activated by growth factors and will further play a key role in CRC development [60]. The Notch pathway can be found in many different cancer tissues, promoting or inhibiting cell proliferation and cell death, thereby interfering with the normal life of cells [61]. Angiogenesis is a process during which endogenous local or systemic chemical signals coordinate the functions of smooth muscle cells and endothelial cells to repair damaged blood vessels [62]. Tumor growth and metastasis depend heavily on angiogenesis [63], and specific tumor cells produce angiogenic and antiangiogenic proteins to stimulate and suppress angiogenesis, respectively [64]. The occurrence, progression, and metastasis of CRC heavily rely on angiogenesis [65]. In summary, these evidences all indicate that CAS15 affects the occurrence of cancer through the significant enrichment of the above tumor-related pathways.

## 5. Conclusion

Overall, our study identified six CASC-characterized hub lncRNAs in CRC, which may be important diagnostic biomarkers in CRC. However, this study still has limitations; this study is a bioinformatic data analysis based on data from public databases, and the clinical value of the hub lncRNAs relies on the next step of a multineutral big data study for further confirmation, which is also the goal of our subsequent study. In addition, the hub lncRNA–protein interaction network identified in this study also requires in-depth in vivo and in vivo experimental studies to study the exact molecular mechanisms involved. Finally, future studies could further deepen the understanding of the tumor immune microenvironment through more immune cell subpopulation analyses and exploration of tumor immune escape mechanisms, which would help to more comprehensively reveal the role of signature lncRNAs in immune regulation and provide a new theoretical basis for immunotherapy.

## Data Availability

The datasets generated and/or analyzed during the current study are available from the corresponding authors upon reasonable request.

## Nomenclature

lncRNAs: long noncoding RNAs
CRC: colorectal cancer
DEGs: differentially expressed genes
AUC: area under the curve
CASC: cancer susceptibility candidate
TNM: tumor node metastasis
ROC: receiver operating characteristic
PCA: principal component analysis
qRT-PCR: quantitative reverse-transcription polymerase chain reaction
MSigDB: Molecular Signatures Database
ssGSEA: single-sample gene set enrichment analysis
MDSCs: myeloid-derived suppressor cells
TME: tumor microenvironment
TIL: tumor-infiltrating lymphocyte
EMT: epithelial–mesenchymal transition
