# Supplementary material for: A Study to Evaluate the Potential Role and Clinical Application Value of Long Noncoding RNA CASC Family Members in Colorectal Cancer Based on Transcriptomic Data
Source: Int J Genomics. 2025 Jul 24;2025:3881424. doi: 10.1155/ijog/3881424 (PMC12313387; doi:10.1155/ijog/3881424)
Supplement: Supporting Information — Additional supporting information can be found online in the Supporting Information section. Table S1: Primers for qRT-PCR. [file 3881424.f1.docx]

Table S1 Primers for qRT-PCR.

| Gene | Primers (5’-3’) | |
| --- | --- | --- |
|  | Forward | Reverse |
| CASC15 | AGCACATAACTGAGAAGGTT | GTTCAGTGTTTCACTCATCA |
| CASC8 | AAAAGCAAGAAGAGAGACCT | AGATAACTAGTGCTCCTGGT |
| CASC9 | ATCATGGGACTCATATTACC | CCAGTTGAAAGGTTCTTCTA |
| CASC19 | GAAGGCTTCCTAAAGAGATA | GCTAGAGTGTTCTCCTGTTC |
| CASC16 | AAGAAAGTACTGGGTCTTCA | GGAGACAAAGGTAGTAATGG |
| CASC18 | TTTTCAGACTCCCTGTAACT | CATATGCCACACTCAGATAG |
| GAPDH | CTTCATTGACCTCAACTACA | AGGCTGTTGTCATACTTCTC |
